# Simulations of blood as a suspension predicts a depth dependent hematocrit in the circulation throughout the cerebral cortex

**DOI:** 10.1371/journal.pcbi.1006549

**Published:** 2018-11-19

**Authors:** Grant Hartung, Claudia Vesel, Prenlin Morley, Ali Alaraj, John Sled, David Kleinfeld, Andreas Linninger

**Affiliations:** 1 Department of Bioengineering, University of Illinois at Chicago, Chicago, IL, United States of America; 2 Department of Neurosurgery, University of Illinois at Chicago, Chicago, IL, United States of America; 3 Department of Medical Biophysics, University of Toronto, Toronto, Canada; 4 Department of Physics, University of California, San Diego, La Jolla, CA, United States of America; University of Western Ontario, CANADA

## Abstract

Recent advances in modeling oxygen supply to cortical brain tissue have begun to elucidate the functional mechanisms of neurovascular coupling. While the principal mechanisms of blood flow regulation after neuronal firing are generally known, mechanistic hemodynamic simulations cannot yet pinpoint the exact spatial and temporal coordination between the network of arteries, arterioles, capillaries and veins for the entire brain. Because of the potential significance of blood flow and oxygen supply simulations for illuminating spatiotemporal regulation inside the cortical microanatomy, there is a need to create mathematical models of the entire cerebral circulation with realistic anatomical detail. Our hypothesis is that an anatomically accurate reconstruction of the cerebrocirculatory architecture will inform about possible regulatory mechanisms of the neurovascular interface. In this article, we introduce large-scale networks of the murine cerebral circulation spanning the Circle of Willis, main cerebral arteries connected to the pial network down to the microcirculation in the capillary bed. Several multiscale models were generated from state-of-the-art neuroimaging data. Using a vascular network construction algorithm, the entire circulation of the middle cerebral artery was synthesized. Blood flow simulations indicate a consistent trend of higher hematocrit in deeper cortical layers, while surface layers with shorter vascular path lengths seem to carry comparatively lower red blood cell (RBC) concentrations. Moreover, the variability of RBC flux decreases with cortical depth. These results support the notion that plasma skimming serves a self-regulating function for maintaining uniform oxygen perfusion to neurons irrespective of their location in the blood supply hierarchy. Our computations also demonstrate the practicality of simulating blood flow for large portions of the mouse brain with existing computer resources. The efficient simulation of blood flow throughout the entire middle cerebral artery (MCA) territory is a promising milestone towards the final aim of predicting blood flow patterns for the entire brain.

## Author summary

The brain’s astonishing cognitive capacity depends on the coordination between neurons and the cerebral circulation, a system known as the *neurovascular unit*. The spatial and temporal coupling between these two networks is the object of intense research. However, the concise anatomical description of the cerebral circulation has so far been intractable. This paper introduces a methodology for the *in silico* creation of realistic models for the entire cerebral circulation. This innovation incorporates topological data from several neuroimaging modalities covering three lengths scales as input into a computer algorithm, which assembles anatomically accurate circulatory networks. When simulating blood flow as red blood cells suspended in plasma for experimental and synthetic cortical network models, we discovered that red blood cells tend to be more concentrated in deeper layers of the cortex compared to the surface. RBC fluxes are more homogenous in deeper layers. The phenomenon of depth dependent red blood cell supply supports the notion that the intricate architecture of the cortical microcirculation serves a *self-regulating* function to maintain uniform oxygen perfusion to neurons. We also demonstrate the practicality of predicting blood flow patterns for the entire brain with existing computer power.

## Introduction

Metabolic activity of the brain is controlled by a complex system of neuroreceptors, small molecular regulators such as nitric oxide, hormones and proteins. The supply, clearance, and balance of metabolites, oxygen, glucose and waste are controlled by the cerebral circulation which is coupled with the cerebrospinal and interstitial fluid (CSF/ISF) subnetworks [1,2]. The coordination between oxygen extraction and increased cerebral blood flow after neuronal firing has garnered intense research interest in blood oxygen-level dependent (BOLD) signal, which is the basis of functional magnetic resonance imaging (fMRI). Recent work [3] has begun to quantify the microvascular origin of the BOLD fMRI signal in a microsection of a mouse brain. The study integrated state-of-the-art neuroimaging of anatomical spaces, tissue oxygen tension measurements and a mechanistic model of blood-bound oxygen supply to convert changes in cerebral blood flow and oxygen extraction into synthetic BOLD signals using Monte Carlo simulations. The main achievement was a successful first principles correlation between measured oxygen and cerebral blood flow (CBF) levels generating fMRI signals.

A recent paper from our group [4] aimed at widening the spatial coverage of coupled blood flow and oxygen simulations. Our model also offered detailed saturation and dissociation kinetics of plasma and red blood cell-bound oxygen, endothelial mass transfer and tissue oxygen extraction. Our study quantified vascular network effects by coupling biphasic (= suspension of red blood cells in plasma) hemodynamics and nonlinear blood rheology with oxygen kinetics. In addition, the number, distribution and position of neuronal and glial cell nuclei were acquired in a sizable section (~1x1x1 mm^3^) of vibrissa primary sensory cortex. We also predicted oxygen saturation in arterioles, capillaries and veins within experimental error bounds. By adopting a probabilistic approach to account for mitochondria respiration associated with specific neuronal and glial somata, the model was used to compute subcellular oxygen gradients between the extracellular matrix, the cytoplasm and individual neuronal/glial mitochondria. The remaining open question concerns the spatiotemporal coordination inside the neurovascular unit.

### Regulation

There is agreement that the neurovascular unit locally controls the cerebral blood flow response. Yet, oxygen supply exceeds the metabolic demand of neuronal activation for reasons that still remain uncertain [5]. Because of the massive size of the mammalian brain with its immense number of neurons and capillaries, the precise temporal and spatial coordination among cellular components still eludes exact physiological description. For example, studies suggest that functional hyperemia causes local neuronal metabolism increase of 5%, which in turn augments local blood flow by 30% up to almost 130% of base line perfusion [6]. However, the exact timing, regulation, and extent of dilation in individual spatially distributed vascular compartments during functional hyperemia are still being investigated [3,7–9].

The cerebral circulation also exercises a second blood flow control mechanism known as cerebral autoregulation [10–17]. Clinical observation [11] suggests that the total cerebral blood flow (CBF) remains constant over a wide range in perfusion pressure (±50 mmHg, ±6666 Pa). Many excellent contributions [18–20] correctly attribute the constancy of cerebral blood supply to global resistance adjustments. Yet, the involvement of specific vascular compartments, speed and spatial coverage of local vasodilatory/vasoconstrictive districts remains elusive [11,18–20]. Moreover, quantification of network effects and control principles among vascular compartments requires an anatomically accurate mathematical model of the cerebral circulation.

Propelled by the advances in neuroimaging, several groups have begun to integrate medical image data with large-scale computer models [3,4,9,21–23]. Generally, these efforts fall into two types. One type adopts a reductionist approach using simplified networks to highlight global blood flow distribution patterns [7,24–28]. The second type follows a bottom-up strategy which aims at replicating relevant microcirculatory components down to the level of the cellular ensemble. Noteworthy examples include quantifying the neurovascular coupling in functional hyperemia [3], analysis of pressure drop dependence on cortical depth [22], predictions of blood flow control by intra-cortical arterioles [9], and cortical oxygen distribution [29,30]. The ultimate goal of bottom-up models is *a hemodynamic simulation of the entire brain*, yet virtual circulation models of the whole brain have been perceived as intractable due to size and nonlinearity of the mathematical coupling between blood flow and oxygen kinetics [24].

This manuscript will introduce a computational procedure that integrates multimodal neuroimage data covering different length scales into a unified virtual representation of the murine cortical circulation. Two-photon imaging provides data for the reconstruction of capillary networks. High resolution micro computed tomography (μCT) imaging is used to capture the connectivity between main arterial branches and pial blood vessels. The morphometrics of the micro, meso- and macro-scale vascular models have been statistically analyzed in order to synthesize virtual blood flow networks with anatomically equivalent statistics, but without being confined to the limited field-of-view or resolution of imaging modalities.

The aim of this paper was to quantify network effects of uneven red blood cell distribution in the cerebral circulation. Although uneven red blood cell distribution also known as plasma skimming can be observed in single bifurcations, neuroimaging of the entire cerebral circulation has so far not been accomplished. To overcome this shortcoming, we integrated physiological data from several neuroimaging modalities covering three different lengths scales. Massive computer simulations of large microcirculatory networks of the murine primary cortex revealed a trend of depth-dependent hematocrit, which is a significant finding indicating that the intricate architecture of the cortical microcirculation serves a self-regulating function to maintain uniform oxygen perfusion.

## Results

### Morphometrics

We first assessed morphometrics of experimental data obtained from murine primary somatosensory cortex samples (N = 4, E1.1-E4.1). The indexing and naming scheme for the data sets is listed in Table 1. The total microvascular segment count was 24,669±9,594 splines. An important property is that all four original two-photon laser scanning microscopy (2PLSM) data sets contained blood vessels that divide into more than two daughter branches (multifurcations). Specifically, the four data sets contained 654, 725, 1686, 1440 multifurcations, respectively.

**Table 1:** Topological feature comparison between experimental 2PLSM and synthetic data sets.

	Experimental 2PLSM (N = 4)	Synthetic data sets (N = 60)
Data name (labels)	E1.1, E2.1, E3.1, E4.1	S{1,2,3,4}.1–15
Number of splined segments (Nsgm)	24,669 ± 9594	24,679 ± 8389
Segments per pial surface (Nsgm/mm^2^)	16,704 ± 2816	16,710 ± 2464
Bifurcations	14,842 ± 5652	16,451 ± 5592
Multifurcations	1172 ± 584	<250
Length (m)	1.38 ± 0.47	1.33 ± 0.47
Intravascular volume (nL)	24.3 ± 12.0	27.0 ± 6.1
Blood-brain barrier surface area (mm^2^)	16.3 ± 6.3	17.7 ± 5.4
Coverage of pial surface area (mm^2^)	1.6 ± 0.3	1.77 ± 0.5
Tissue volume (mm^3^)	2.2 ± 0.8	2.2 ± 0.7

Statistics on cumulative metrics including vascular surface area, path length and luminal volume are compiled in Table 1. Although the data originate from the same cortical region, there are subject-specific variations between different specimens. There was higher variability in the low end of the vascular diameter spectra, because unavoidable uncertainty affects the thinnest vessels close to image resolution threshold as observed previously [31]. We also estimated the surface area to tissue volume ratio of the blood-brain-barrier (BBB) of the microvascular network as 8.8±1.1 mm^2^ vasculature/mm^3^ tissue. This number was obtained by summing the (endothelial) surface area of the capillary bed; this estimate compares to experimental values of the BBB surface of about 10-17 mm^2^/mm^3^ in humans [32–34].

### Synthetic data sets

A *modified constructive growth algorithm* (mCCO [30]) was used to create 60 synthetic data sets (S1.1-S4.15) of the murine primary sensory cortex. For each of the four experimental data sets, 15 clones with statistics matching closely their experimental original were created, so that the S1.1–15 series matched the original E1.1, and S4.1–15 matched data set E4.1. Artificial networks smoothly connect arterial vessels through the capillary bed to the veins without gaps or the need to insert artificial segments as observed with other methods [27]. In addition, since blood vessels are not exactly straight, realistic tortuosity values were imposed by a Bezier spline-based technique described previously [30]. Moreover, at the boundaries of the synthetic data sets neither pial surface vessels, nor deeper laying arterioles, capillaries, or venules were severed or had to be pruned thanks to the precise geometric control of the vasogenic growth algorithm. Artificial network growth took less than five minutes for each dataset on a personal computer.

### Branching patterns

**Fig 1:**
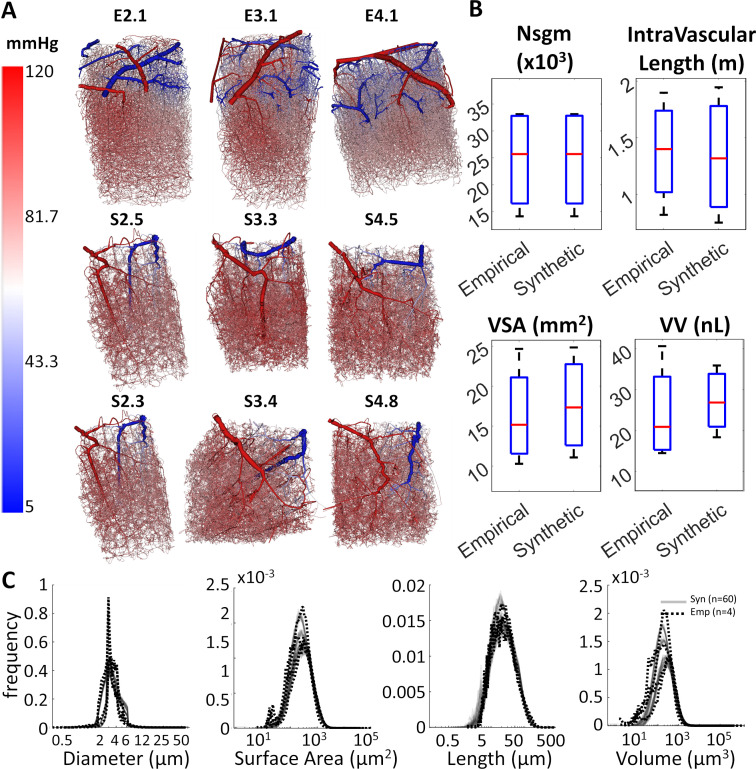
Morphometric comparison between experimental and synthetic microcirculatory networks from the murine vibrissa primary sensory cortex. (A) Three 2PLSM experimental [35] data sets (E2.1, E3.1, E4.1, three shown out of four) are compared to synthetic (S2.5, S3.3, S4.5, S2.3, S3.4, S4.8) microcirculatory networks (six shown, out of sixty total). (B) Cumulative microcirculatory morphometrics for experimental (N = 4) and synthetic (N = 60) networks (segment number-Nsgm, intravascular length, vascular surface area-VSA, and vascular volume-VV are statistically similar, p>0.05 in all cases using one-way ANOVA). (C) Probability density functions show that the synthetic data sets are not identical, but match the topology of experimental data sets. Taken together, the morphometric analysis shows that experimental and synthetic networks are statistically equivalent.

We also compared morphometrics of experimental (N = 4) against synthetic vibrissa primary somatosensory cortex data sets (N = 60, S1.1-S4.15). No discernible feature differences can be inferred from visual inspection as shown for three experimental (E2.1, E3.1, E4.1) and six synthetic data sets (S2.5, S3.3, S4.5, S2.3, S3.4, S4.8) in Fig 1A. Total count amounted to 24,679 ± 8389 spline segments and 16,451 bifurcations. Spline segments were defined as tubular connections (splines) between branching points (bifurcations or multifurcations). This counting method ensured that the final tally is independent of image grid resolution or number of segment sub-partitions. The comparison of cumulative properties and probability density functions shows excellent agreement between the experimental and synthetic networks as seen in the plots Fig 1B and 1C. The synthetic networks are different realizations, but statistically equivalent replica (clones) of the original image samples.

### Network effects in large-scale models

The nonlinear biphasic blood flow, pressure and hematocrit equations for all four experimental networks and all sixty synthetic networks converged within five minutes [29]. Results were visualized with 3D rendering software Walk-In Brain developed at our institution [36,37]. Path analysis was conducted based on flow trajectories traversing the network along streamlines. Biphasic blood flow and network effects determining blood pressure and hematocrit distribution through large experimental (N = 4) and synthetic (N = 60) networks perfusing a large portion of the cortex were studied.

**Fig 2:**
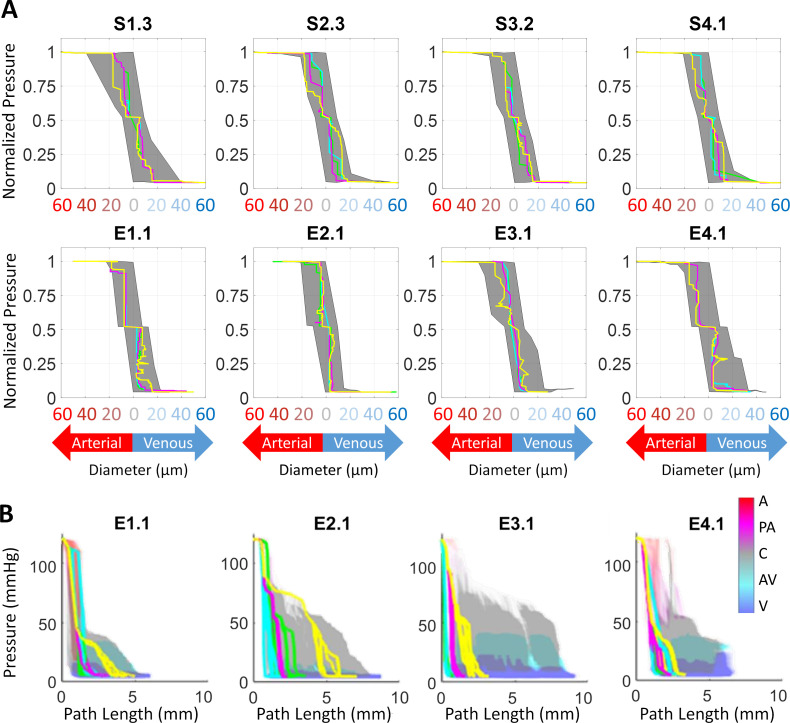
Predictions of hemodynamic states in primary cortex simulations show large variations due to network architecture. (A) Path analysis (N = 2,300–22,052 paths per dataset) of blood pressure as a function of diameter. (It should be noted that some paths in the experimental data sets e.g. E1.1 exhibit zigzagging which is probably due to uncertainty in the diameter information). (B) Blood pressure as a function of path length in four microcirculatory data sets. Representative path trajectories have been plotted in green, blue, magenta and yellow (A-arteries, PA-penetrating arteries, C-capillaries, AV-ascending venules, V-veins).

Typical pressure distributions along the microcirculatory network hierarchy are shown in Fig 2. Pressure drop trajectories through the microcirculation showed patterns consistent with experimental data [38–40]. Results of the path analysis in Fig 2 also depict the wide variations of hemodynamic states when blood traverses the dense microcirculatory network from the pial surface vessels through penetrating arterioles into the capillary bed and finally back to the collecting veins. The trajectories of individual paths (green, blue, magenta and yellow) display wide variability of hemodynamic states along the flow direction. Flow analysis reflected that a perfusion pressure drop in the microcirculatory networks from 120 to 5 mmHg (15,999-667 Pa) resulted in a mean tissue perfusion of 68.9 ml/100g/min (11 ∙ 10^−6^ m^3^/kg/s) which is within experimentally observed ranges [41,42].

### Path analysis of hematocrit and layer dependence

**Fig 3:**
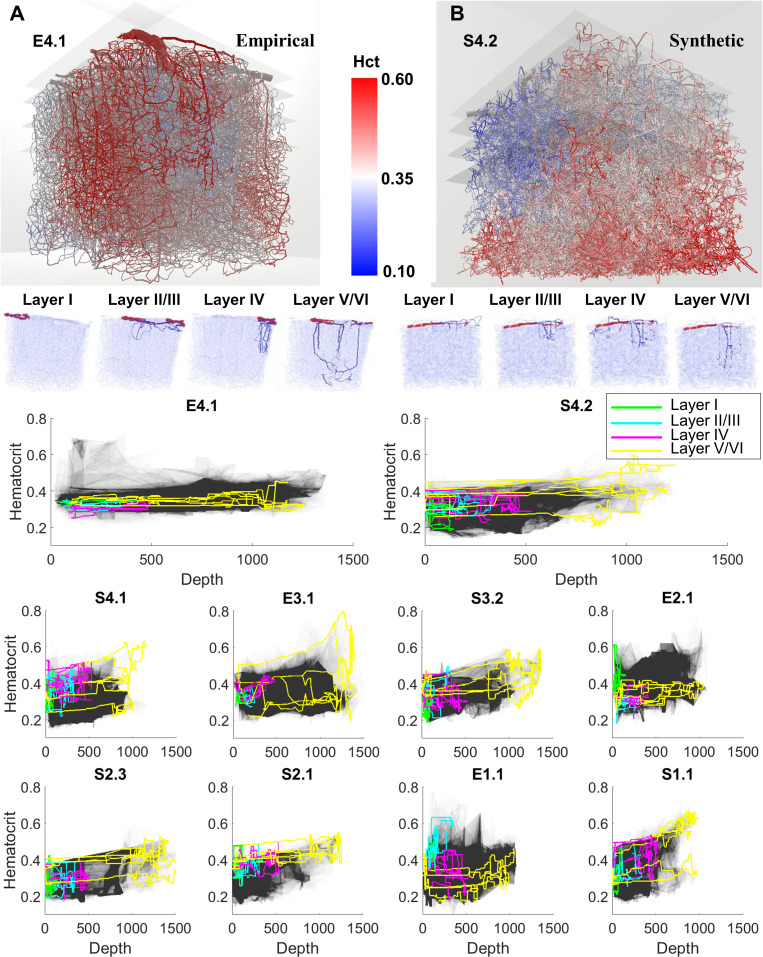
Depth dependent path analysis of hematocrit trajectories through the cortex. The visualization of the hematocrit field in three dimensions shows a higher (red) level of discharge hematocrit in the deeper segments than in segments closer to the pial surface. This trend is observed in experimental (A, E4.1) and synthetic (B, S4.2) networks. Plots for all paths (N = 2,300-22,052 paths per dataset) trace the grayed region, depicted here for experimental data set E4.1 and synthetic data set S4.2. For better visibility, representative paths descending to different depths are shown with color coding by layer (I-VI). Deeper reaching paths (layer V/VI-yellow) tend to carry higher hematocrit levels than shallower paths (layer I-green). (C) Additional data sets show consistently depth dependent hematocrit in synthetic (N = 5, S1.1, S2.1, S3.2, S2.3, S4.1) and experimental (N = 3, E1.1, E2.1, E3.1) networks (eight examples depicted).

**Fig 4:**
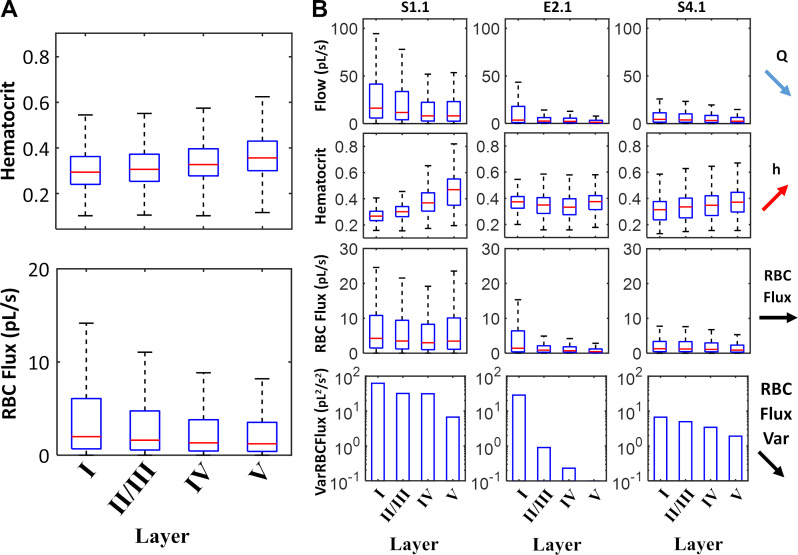
Statistics of hematocrit distribution and RBC fluxes in cortical layers of cerebral microcirculatory networks. (A) Statistics over an ensemble of experimental (N = 4) and synthetic (N = 12) data sets show higher discharge hematocrit in deeper segments (N = 1,234,412 with p<0.01 using one-way ANOVA). The median (red line), 25-75^th^ percentile (blue box) and limits (black lines, excluding outliers) of hematocrit in all blood vessels for each layer obtained for all data sets. (B) Statistics in three individual data sets (S1.1, E2.1, S4.1, N = 59830, 94842, and 108833 respectively, p<0.01 in all cases). In all case, layer V has higher hematocrit levels than the layers closer to the cortical surface. In general, shorter surface paths (layer I) tend to have higher flow rate, *Q*, but lower hematocrit levels, *h*. The total red blood cell flux (RBC) is rather uniform for all cortical layers, because the flow effect (*Q*, lower in deep layers) and hematocrit (*h*, higher in deep layers) balance each other out. The variance of the RBC fluxes, *VarRBCFlux*, decreases with depth; accordingly there is more homogenous RBC flux distribution in deeper layers.

We further inspected the RBC flux distribution as a function of network hierarchy (= vascular) and position inside the cortical hierarchy (= neuronal). The results were acquired for both empirical and synthetic data sets. Two representative specimens are highlighted in Fig 3A and 3B; eight more examples are displayed in Fig 3C. Typical paths belonging to different cortical layers are color coded in Fig 3. Flow paths were generated by tracing the flow from arterial inlet nodes downstream through the capillary bed until reaching a venous outlet. Paths were sorted according to their tissue supply function as follows: a path depth label equal to the cortical depth of the deepest segment was assigned to each flow path. Thus, all paths were uniquely ordered within a spectrum of shallow to deep reaching paths according to the neuronal layer (I-VI) hierarchy in agreement with previously reported values [35,43,44]. Fig 3 depicts hematocrit values along representative paths in shallow (layer I-green) and deeply penetrating paths (layer V/VI-yellow). Along each path and between different paths there is high variability along the flow direction. For example, discharge hematocrit in data set S1.1 reaches values as high as h_max_~0.7, and as low as h_min_~0.18. However, there is an overall trend of higher hematocrit being carried to lower cortical levels (layer-V/VI paths). The trend of relatively higher hematocrit, h, conveyed to deeper tissue layers (p-value<0.01, using one-way ANOVA test in MatLab) was observed consistently in all experimental and synthetic data sets. The bulk flow, Q, showed the opposite trend; it was reduced in segments of deeper layers which are connected by longer paths as is summarized in Fig 4. In contrast to bulk flow and hematocrit, the RBC flux (= volumetric flow rate of the RBC phase) exhibited weak layer dependency, it was almost constant irrespective of the cortical depth. We also observed that the variance of capillary RBC fluxes decreased with cortical depth, thus RBC fluxes in deeper layers show lower variability than paths on the surface. Taken together, biphasic blood rheology and network effects seem to induce depth dependent hematocrit supply to the cerebral cortex which leads to more homogenized RBC fluxes in deeper layers (= lower variance in RBC fluxes). Further analysis of diameter dependence on hematocrit confirmed the high degree of hematocrit variability across the diameter spectra as previously observed [4] (S1 Supplement).

The agreement between the simulation results obtained for experimental and synthetic data confirms that the synthetic networks are hemodynamically equivalent to the experimental networks. The satisfactory match in morphometrics and hemodynamics between experimental and synthetic data justifies the extension of network synthesis to large anatomical regions as described next.

### Extension to brain-wide hemodynamic simulations

**Fig 5:**
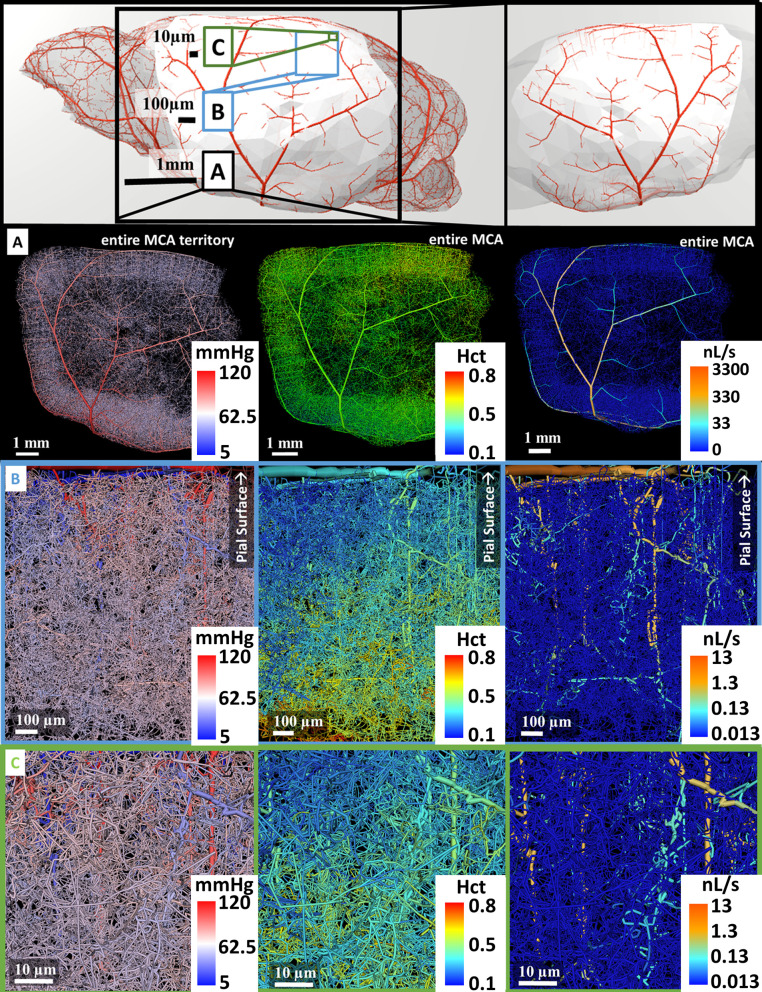
Schematic of multiscale biphasic blood flow simulations in the arterial side of the MCA territory. The large-scale cerebrocirculatory model connects the Circle of Willis to the territory of the middle cerebral artery with its complete pial arterial network and microvasculature. Simulation results show snapshots of pressure distribution, flow rates, and hematocrit at three length scales (1mm, 100μm, 10μm). The three views roughly correspond to the resolution of several imaging modalities: top layer, A, depicts the major arteries and anatomical features at the millimeter scale as seen in μCT imaging; the middle layer, B, shows arterioles at the micron range; the bottom layer, C, reaches cellular resolution as seen in 2PLSM or with confocal imaging.

Vascular networks covering the circulation of the entire MCA territory were generated with the help of our modified CCO (mCCO) algorithm as described in Gould et. al [30]. The mCCO algorithm was launched with the MCA M1 as the first segment. The location of the MCA territory within the context of the mouse cortex is shown in Fig 5 top-row. Sequentially, more segments were added at the cortical surface depicted in Fig 5 top-row, while minimizing the vascular tree volume subject to blood flow constraints. Thus, gradually the algorithm generated all arterial branches of the pial network on the cortical surface. Then, it was directed to proceed with penetrating arterioles and microcirculatory growth to a depth of approximately 1 mm below the pial surface, until a preset vessel density was reached. At each step of the segment generation, connectivity and bifurcation position were optimized to obtain minimum tree volume. The diameters of the network branches were recursively recomputed in accordance with hemodynamically-inspired principles [45]. The total number of splined segments in the artificial MCA territory was 993,185. This was roughly 60 times the number of segments in the cortical samples.

The topology of the synthetic MCA territory resembled maps available in mouse atlases [46,47]. Branching density and pattern of the pial arteries as well as the number of penetrating arterioles was within ranges of the reconstructed sets of μCT images as listed in Table 2. Detailed views in Fig 5 show pial, microcirculatory and individual capillary scales illustrating different aspects of the massive network model covering three length scales ranging from the MCA M1 segment with a diameter [48] of 142 µm down to the capillary bed [35], d<6 µm. Morphometrics of the synthetic MCA networks are summarized in Table 2. Fig 5A–5C depicts the pressure, flow and hematocrit field from the outflow of the Circle of Willis (MCA M1), down to the smallest capillaries in the microcirculation. The anatomical detail and branching pattern is depicted for the highly irregular, tortuous microcirculatory network.

**Table 2:** Pial network parameters used in this work in comparison to prior research.

Parameter	Value	Units	Citation
Penetrating arterioles surface coverage	13 ± 3	Nsgm/mm^2^	Nishimura [49]
	13	Nsgm/mm^2^	This work (entire MCA territory)
Average penetrating arterioles diameter	11	µm	Blinder [35]
	11	µm	This work (entire MCA territory)
Larger artery diameter	143 ± 8	µm	Kidoguchi [48]
	142	µm	This work (entire MCA territory)
Mouse brain volume	453 ± 19	mm^3^	Ma [50]
	509 ± 23	mm^3^	Badea [51]
	415 ± 24	mm^3^	Kovačevič [52]
Sagittal Length	13	mm	Kovačevič [52]
	13.7	mm	Diem [53]
	13	mm	Clavaguera [54]
	14	mm	Natt [55]
	13.7	mm	This work (entire MCA territory)
Coronal Height	10	mm	Kovačevič [52]
	8	mm	Diem [53]
	9	mm	Natt [55]
	8.0	mm	This work (entire MCA territory)
Coronal Width	5	mm	Kovačevič [52]
	5.5	mm	Diem [53]
	5.5	mm	Clavaguera [54]
	6	mm	Natt [55]
	5.7	mm	This work (entire MCA territory)
Cortical surface area	380 ± 20	mm^2^	Ma [50] (young mice)
	348 ± 3	mm^2^	Badea [51]
Number of splined segments	993,185	Nsgm	This work (entire MCA territory)
Segments per pial surface	25,144	Nsgm/mm^2^	This work (entire MCA territory)

Nsgm–number of splined segments

### Complete circulation of the MCA territory including arterial and venous side

**Fig 6:**
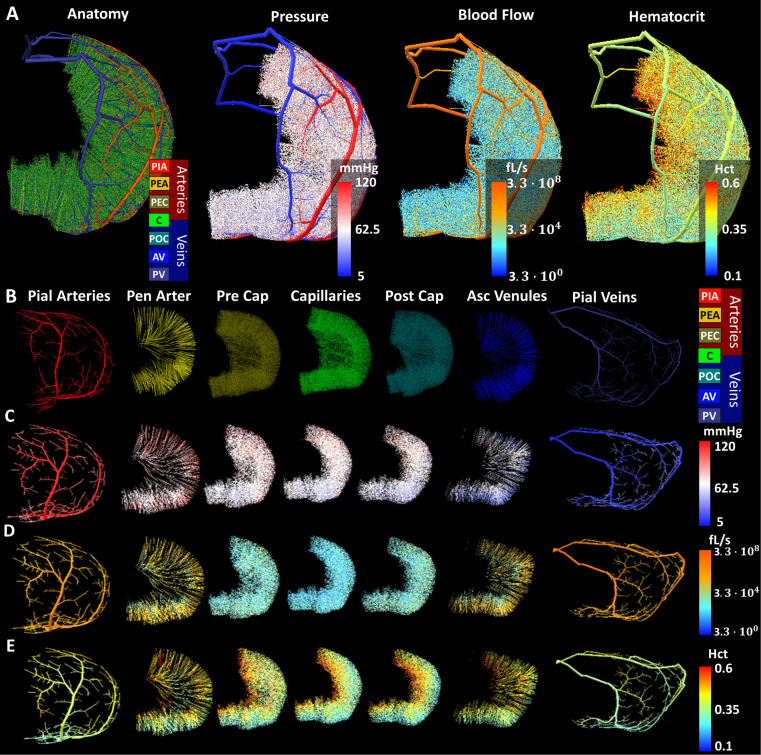
Blood flow of the complete arterial and venous circulation for the MCA territory in mouse. This large-scale model contains the MCA M1 segment branching from the Circle of Willis as inflow and covers the entire territory of the middle cerebral artery with a complete pial network and microvasculature encompassing PIA-pial arteries, PEA-penetrating arterioles, PEC-pre-capillaries, C-capillaries, POC-post-capillaries, AV-ascending venules, PV-pial veins. (A) Three dimensional snapshots of the spatial distribution of anatomical grouping, blood pressure, blood flow (perfusion), and hematocrit. Explosion diagram of anatomical compartments of the angioarchitecture in the MCA territory; color-coding depicts (B) anatomical groups, (C) blood pressure, (D) flow (perfusion) and (E) hematocrit distribution. This large-scale model contains 5,452 spline segments of the pial network, 27,374 splines encompassing penetrating arterioles and ascending venules, and 960,359 capillaries. The volume of the pial arteries is 143 nL (16.5%), penetrating arterioles is 75.1 nL (8.7%), precapillary arterioles is 101.9 nL (11.8%), capillaries is 96.8 nL (11.2%), post-capillary venules is 100.7 nL (11.6%), ascending venules is 75.8 (8.7%) and pial veins including portions of the superior sagittal sinus is 273.8 nL (31.5%).

The simulation of the entire MCA territory included the compartments of pial arteries, penetrating arterioles, pre-capillaries, capillaries, post-capillaries, ascending venules and pial veins. To complete the MCA circulation, the venous tree including venules was synthesized in reverse and connected to the capillary bed as described previously [30]. Fig 6 depicts the distribution of pressure, flow and hematocrit throughout the MCA territory. Fig 6A shows comprehensive three-dimensional maps of the anatomical hierarchy, pressure distribution, blood flow in the MCA territory, and uneven biphasic hematocrit. Fig 6B–6E highlights the anatomical grouping, pressure, flow, and hematocrit distribution throughout individual compartments. In these views, explosion diagrams separating the anatomical groups (pial arteries, penetrating arterioles, pre-capillaries, capillaries, post-capillary venules, venules and pial veins) were used to better delineate the hemodynamic states in each group. Visual inspection of the microcirculatory compartments (pre-capillaries, capillaries, and post-capillaries) depicted in Fig 6E reveal higher hematocrit levels in deeper cortical layers than on the surface.

### Blood flow

Simulations conducted for the entire circulation on the MCA territory required boundary conditions at only two points; MCA M1 arterial blood pressure (p = 120 mmHg, 15,999 Pa), hematocrit level (h = 0.35), and venous outlet pressure (p = 5 mmHg, 667 Pa). The solution encompassed blood pressure, flow and hematocrit for 5452 pial vessels, 27,374 segments perpendicular to the pial surface, and 960,359 capillaries of the entire center MCA territory, for a total of 993,185. In total, the proposed iterative method succeeded in bringing to convergence a total of 2,648,853 equations for biphasic blood flow.

**Fig 7:**
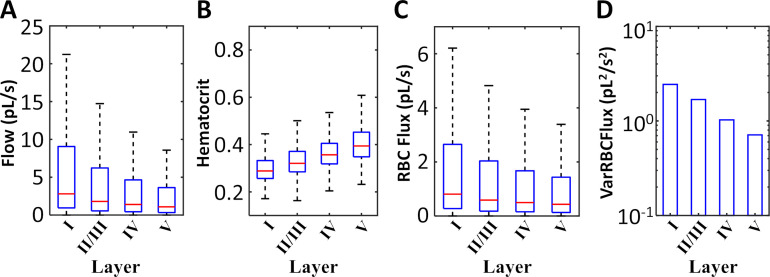
Depth dependence of hematocrit on total blood flow in the center MCA territory. Biphasic blood flow simulations for the entire MCA territory were analyzed statistically to illustrate depth dependence of hemodynamic states. In a large subsection cut out of the center MCA cortical vasculature (with volume = 4.1 mm^3^ and surface area = 4.2 mm^2^ which equals 11% of the MCA territory), 188,865 microvascular segments in layers I-V were assessed. (A) Blood flow slightly decreases in deeper paths (p < 0.01 using one-way ANOVA test). (B) Hematocrit increase along deeper cortical layers (p < 0.01, one-way ANOVA test). (C) The product of bulk blood flow and hematocrit gives the RBC flux, which is almost constant with a mild decrease with depth (p < 0.01 using one-way ANOVA test). (D) The variance of RBC flux (*VarRBCFlux*) decreases with depth, so RBC flux distribution is more homogenous in deeper layers than close to the surface.

The predicted perfusion rate for the MCA territory was 50 ml/100g/min (= 8.3 ∙ 10^−6^ m^3^/kg/s) which is in agreement to literature ranges [41,42] of 40–163 ml/100g/min (= 6.7–27.2 ∙ 10^−6^ m^3^/kg/s). The trend of higher hematocrit levels in deeper cortical layers seen in the smaller cortical samples was also confirmed in the massive simulations for the MCA territory as shown in Fig 7.

It should be noted that the simulations showed virtually no boundary effects in the center of the MCA territory where the primary sensory cortical samples were located. The suppression of boundary effects that can be achieved by large-scale simulation is extremely important for simulating hemodynamic blood flow control such as it occurs in functional hyperemia or under autoregulatory control. A full simulation of the entire MCA territory (arterial and venous side) required 65 iterations and ~2 hours on multicore workstations.

## Discussion

### Morphometrics

We performed multiscale morphometric analysis of the cerebral circulation in mouse over three length scales. On both the macro and the mesoscale, statistical data for the Circle of Willis, the middle cerebral artery and its pial arterial network were extracted from high quality micro-CT (µCT) data [56]. Microcirculatory morphometrics were acquired by two-photon imaging (2PLSM) delineating the micro-angioarchitecture down to the level of individual capillaries for sizable sections (~1x1x1 mm^3^) of the vibrissa primary sensory cortex. There were statistical differences between the 2PLSM microcirculatory data sets especially in the diameter information as can be expected from a high resolution analysis of cortical microcirculatory networks. However, these variations did not significantly alter hemodynamic flow patterns. The morphometrics (arterial, capillary and venous segment number, connectivity and branching patterns, probability density functions for length, diameter and surface area spectra) informed a synthetic vascular growth algorithm. Because the statistics (e.g. segment numbers) could directly be input into the mCCO algorithm, we were able to create 15 synthetic replica for each of the four data sets. In total, we synthesized artificial vascular networks (N = 60) with morphometrics and blood perfusion patterns that are statistically equivalent to the experimental data. The wealth of experimental and synthetic data used in this study provided a testbed for hemodynamic analysis of biphasic blood flow through the cortical microcirculation.

### Blood flow

Hemodynamic simulations were performed using computer algorithms described and tested extensively [29]. We performed biphasic blood flow simulations on both experimental (N = 4) and synthetic microcirculatory networks (N = 60). Simulation results predicted patterns of blood flow, pressure and hematocrit within ranges currently known from experiments. Even though our blood flow computations are deterministic [4,29], computed hemodynamics states varied widely within the labyrinth of paths traversing the microcirculation. We pinpointed randomness of the angioarchitecture as the origin of the wide range of predicted hemodynamic states. The finding of variability in hemodynamic states due to network architecture is significant, because it suggests that there are no characteristic properties (e.g. average hematocrit, mean capillary pressure) that would justifiably represent a typical physicochemical state of a microvascular compartment (arterioles, capillary bed, venules). It also explains why idealized trees such as binary ordered hierarchical graphs [26] are unsuitable surrogates for microcirculatory flow networks, because their regular and symmetric branching patterns lack the randomness in network topology seen in the murine anatomy. Specifically, ordered trees have equal states in all branches of a given hierarchy, which leads to even hematocrit splits due to symmetry in daughter branching diameters.

Variability in hemodynamic states reported previously [4] has implications for neuroimaging research. Specifically, even exact measurements at an individual point within the limited neuroimaging field of view (e.g. ~1 mm^2^ surface in two-photon images) would be prone to exhibit wide variations. The patchiness (variability) obtained by image acquisition at a single point cannot be overcome by more accurate imaging. Instead, an effective response to counteract variability due to network randomness is to adopt imaging protocols that emphasize spatially distributed samples over point measurements. In other words, measurements intended to infer global trends necessitate spatially distributed samples. Specifically, point observations acquired for single blood vessels can be expected to exhibit wide variations due to network effects, even if measurements are precise.

### Hematocrit

**Fig 8:**
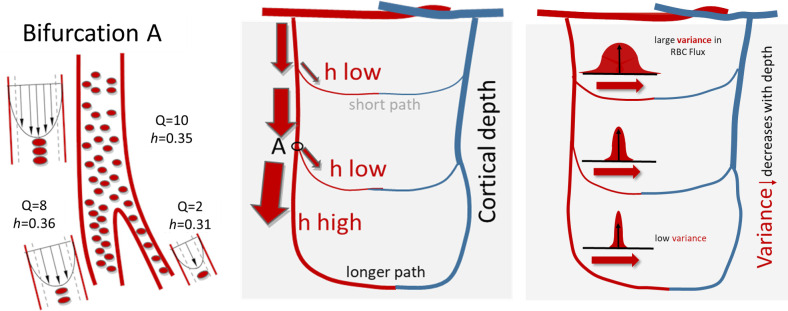
Schematic of the depth dependent hematocrit network effect. At the microcirculatory level, blood is a suspension of red blood cells in plasma with a thin boundary layer close to the vascular wall which contains little or no red blood cells. When red blood cells suspended in plasma flow through a bifurcation of a penetrating artery, they tend to concentrate in the thicker daughter branch, while the thinner side branch syphons a comparatively higher fraction of plasma from the cell free layer near the wall of the parent branch. This effect is known as *plasma skimming*. When plasma skimming repeats over many bifurcations of the cortical microcirculation, deeper reaching paths through the capillary bed tend to have higher hematocrit than surface paths. Longer path length incurs higher flow resistance leading to less bulk flow. In effect, total RBC flux, which is the product of hematocrit times flow, is more balanced than if RBC splits were even. The network effect also reduces variability in RBC fluxes, so that deeper layers are more evenly perfused. Our simulations implicated network effects due to biphasic blood rheology for the predicted hematocrit gradients and increased RBC flux homogenization.

Our large-scale computer simulations suggest a depth dependent hematocrit gradient in the cortical blood supply as summarized conceptually in Fig 8. Detailed analysis of the spectrum of individual microcirculatory blood flow paths illuminated a clear trend; namely that deeply penetrating microvessels convey more red blood cells than paths running closer to the pial surface. The observation of higher hematocrit in deeper paths was observed in all simulation experiments for the primary sensory sets (experimental data sets, N = 4; synthetic microcirculatory networks, N = 60 as seen in Fig 4) as well as for the large-scale blood flow simulations covering the entire MCA territory shown in Fig 7. The predicted homogenization effect results in more uniform RBC fluxes, because shorter superficial paths tend to have higher bulk flow, Q, but carry less hematocrit, h. On the other hand, longer deeper penetrating paths have to overcome higher resistance leading to lower flows, but enjoy increased hematocrit as summarized in Fig 4 and Fig 7. As a consequence, this phenomenon also suggests that shorter surface paths which tap into fresh arterial oxygen supply have fewer RBCs, while deeper paths have higher concentrations of RBCs which on average carry lower O_2_ saturation. Another effect of hematocrit gradient is that net oxygen fluxes conveyed to different cortical layers are more evenly balanced than would be the case if RBCs distributed uniformly (no plasma skimming). We also noticed that the variance of RBC fluxes decreased with cortical depth. Accordingly, the distribution of RBC fluxes in deeper layers is more homogeneous than in surface layers. Random network architecture together with non-uniform hematocrit distribution due to the complex biphasic blood rheology seems to be two synergetic factors for ensuring homogenous oxygen supply irrespective of the cortical tissue depth. Since this homogenization effect needs no external feedback, it is plausible to infer that layer dependency of hematocrit and reduction of RBC flux variance serves a self-regulatory mechanism to balance oxygen supply to all cortical layers.

The plasma skimming effect describes a phenomenon seen in microvascular bifurcations (d<300 µm) [57,58] in which thinner side branches syphon disproportionately large amounts of plasma from the parent segment than thicker daughter branches. Our mechanistic simulations illustrate how plasma skimming phenomena apply over thousands of bifurcations and multifurcations in a tortuous vessel network, effectively overcoming the geometrical unavoidability of path length differences as shown in Fig 8.

Our recently developed kinetic plasma splitting model (KPSM) was our choice for computing large-scale network effects in this study. The main critical reasons include: (i) the KPSM split rule is able to handle multifurcations that occur in the murine microcirculatory anatomy (7.1%, 5.9%, 8.9%, 6.7% of all segments had multifurcations in experimental data sets), (ii) its predictions fall within physiologically meaningful property ranges. Specifically, it does not lead to predictions of zero or excessive hematocrit, and (iii) its linear and differentiable mathematical properties guarantee convergence of massive network computations. A full account documenting the KPSM model can be found in S3 Supplement.

### Synthesis

The previously introduced network synthesis used a modified constrained constructive optimization (mCCO) [30] algorithm. The mCCO algorithm originally conceived by Schreiner [45] deploys two very simple principles: (i) minimization of vascular volume, and (ii) hemodynamic flow principle constraints which enforce that the total blood flow entering the network discharges in exactly equal amounts through the terminal outflow segments. Remarkably, this approach builds network structures whose topology resembles vascular network anatomy observed in vivo. One major task consisted of testing whether realistic network representations with arterial-capillary-venous closures could be synthesized with morphometric and hemodynamic properties matching networks acquired with neuroimaging modalities. The results showed that synthetic data (N = 60) created with a modified mCCO algorithm were statistically and hemodynamically equivalent to experimental cortical data sets (N = 4).

### MCA

The hemodynamically inspired vascular growth procedure enabled the construction of realistic representations of the cortical blood supply of the entire MCA territory spanning multiple length scales from the large arteries (mm range) to the smallest capillaries (µm range), and draining through the pial veins (mm range) or three orders of magnitude in length scales. It allowed us to seamlessly integrate state-of-the-art topological data acquired from two entirely different imaging modalities (µCT and 2PLSM) into a single, coherent multiscale representation of the entire MCA territory with unprecedented anatomical detail that includes both the arterial and the venous side of the cerebrocirculation. Because simple, blood flow inspired construction principles are applied at all length scales, the resulting MCA circulation has no discontinuities or gaps between the main cerebral arteries, the pial arterial network, or the microcirculation. Morphometrics, anatomical details such as the shape of the cortical surface and hemodynamic principles, are incorporated at each stage of the growth algorithm. Thus, our proposed methodology may serve as an alternative to the practice of merely stitching together data from different locations or length scales.

The application of biphasic blood flow simulations for the entire MCA territory shows that large-scale blood flow and hematocrit simulations are feasible with existing computer resources. The large-scale simulations confirmed the trend of hematocrit layer dependence predicted for the smaller cortical samples. The massive simulations also elucidate the spatiotemporal coordination between different vascular compartments at different length scales (arteries vs arterioles vs capillary bed). The anatomical detail achieved with the MCA model may serve as a starting point for dynamic simulations that elucidate the involvement of different vascular components in regulating functional hyperemia, autoregulation or collateral blood supply in stroke. Because the network extended over a sizable portion of the mouse cortex, predictions for the center of the primary sensory cortex were free of boundary effects.

### Boundary conditions

The synthetic MCA circulatory network also has the critical advantage that boundary conditions, which have been reported to hamper simulations on thin data sets [9], are applied very far away from the area of investigation. For example, Fig 5 displays typical subsections comparable in size to the 2PLSM data sets which are located far away from the MCA boundaries (MCA M1 segment and veins of the superior sagittal sinus). Thus, in samples situated at the center of the MCA territory, boundary conditions have negligible impact on hemodynamic predictions. The blood flow simulation for the entire MCA territory required only the arterial inlet pressure at the M1 segment and the blood pressure at the venous side.

We point out three additional reasons why the ability to synthesize morphologically and hemodynamically equivalent data sets is significant. (i) Artificial networks continuously connect the arterial side and the venous side without gaps. In 3D neuroimages assembled from two-dimensional image stacks, it is easy to miss segment connections or segments running between two slices. (ii) No segments are severed nor is there a need to prune dangling segments at domain bounds (this cleanup is unavoidable in image reconstructions [3,23]). In particular, fragmentation to pial arteries and many microcirculatory segments running perpendicular to the pial surface lead to boundary effects that can substantially affect predictions [9]. (iii) The most important benefit is the ability to expand the scope of data acquired by neuroimages without being confined to the bounded field-of-view or limited resolution of the imaging modality.

The ability to conduct brain-wide simulations would free the modeler from the burden of making uncertain assumptions at the boundaries of the artificial domain (edge of the image or simulation domain boundary). Because our algorithm succeeded in converging blood flow computations with hematocrit split for the entire MCA circulation in about two hours of CPU time, our group is confident that the proposed computational approach will enable blood flow simulations and oxygen transport on a brain-wide level in the near future.

### Limitations

Despite the evidence for trends such as depth dependent hematocrit, it should be emphasized that individual flow paths may experience substantially weaker or even reverted trends, as can be expected from the inherent randomness of the microcirculatory network architecture.

The 2PLSM technique provided a very detailed inventory of the cortical microcirculation. The four data sets did not include information about the subcortical blood supply to the white matter. White matter subcortical circulation is physiologically separated from the cortical blood supply. Accordingly, we assumed that the white matter supply is hydraulically separated from the cortical blood supply. However, certainty about this point would require a model of both the cortical and the subcortical networks (white matter blood supply). This task is intriguing, but is currently beyond the reach of 2PLSM, which is limited to ~1 mm depth. This is clearly a point for future research, but is currently outside the scope of this paper.

The main finding of depth dependency of hematocrit supply to the cortical layers is the result of a model prediction whose basis rests on experimental observations about plasma skimming and uneven hematocrit splits observed in capillaries outside the brain [59–61]. Therefore, the next logical step is to experimentally verify layer dependent hematocrit with deep imaging such as adaptive optics (AO) two-photon imaging [62]. If experiments confirm depth dependence and homogenization of RBC flux distribution, it would constitute a remarkable mathematical modeling contribution, which actually predicted, instead of merely explained, cortical blood supply. In the adverse case, the model would have prompted the need to revise our understanding of biphasic blood flow rheology as it relates to the cortical microcirculation (= diameter and hematocrit dependent viscosity laws, and hematocrit split rules), since so far it has been assumed that plasma skimming is active in capillaries throughout the entire circulatory system including the brain.

The conclusions about oxygen supply also need to be verified experimentally and computationally. The methods presented previously might be a first step in this direction [4]. However, oxygen predictions require discretization of the extracellular space which can be done in principle using the methods presented in Gould et. al [29], but is beyond the scope of this paper.

### Conclusions

We predicted uneven depth dependent hematocrit distribution due to the complex biphasic blood rheology. Because our simulation did not include external factors such as gravity, we conclude that the result of depth dependent hematocrit arises from the combination of structural and hemodynamic properties of the network. Our findings suggest that network effects due to biphasic blood rheology and randomness of the network architecture are a controlling factor for ensuring adequate oxygen supply irrespective of the cortical depth. Since the observed homogenization of RBC variability requires no feedback, depth dependent hematocrit gradient may serve an important self-regulatory mechanism to balance oxygen supply to all cortical layers.

Uneven distribution of hemodynamic states in the microcirculation as well as the notion of layer-dependent hematocrit also have implications on the interpretation of the fMRI BOLD signal where it is usually assumed that hemodynamic states and hematocrit are homogeneous and evenly distributed throughout the microcirculation. The predictions in this work suggest that focal analysis of the fMRI BOLD signal would be more relevant than assuming global constants for the entire cortex.

We demonstrated that the modified constrained constructive optimization algorithm (mCCO) is successful in synthesizing artificial microcirculatory networks with topological and hemodynamic properties that are statistically equivalent to experimental data sets from different imaging modalities and length scales.

Simulations of the entire MCA circulation, which until recently would have to be considered intractable, are now becoming accessible to rigorous numerical analysis due to stable, efficient and physiologically consistent plasma skimming algorithms implemented on existing computer hardware. The synthesis of anatomically faithful cerebrocirculatory networks with desired topology closes the gap between large-scale blood flow simulations performed on image-derived data sets on one hand, and simulations on purely synthetic data sets on the other.

The successful synthesis of the entire MCA territory with biphasic blood flow simulation constitutes a step towards the ultimate goal of first principle simulations of cerebrocirculatory blood and oxygen distribution patterns for the entire brain.

## Materials and methods

**Fig 9:**
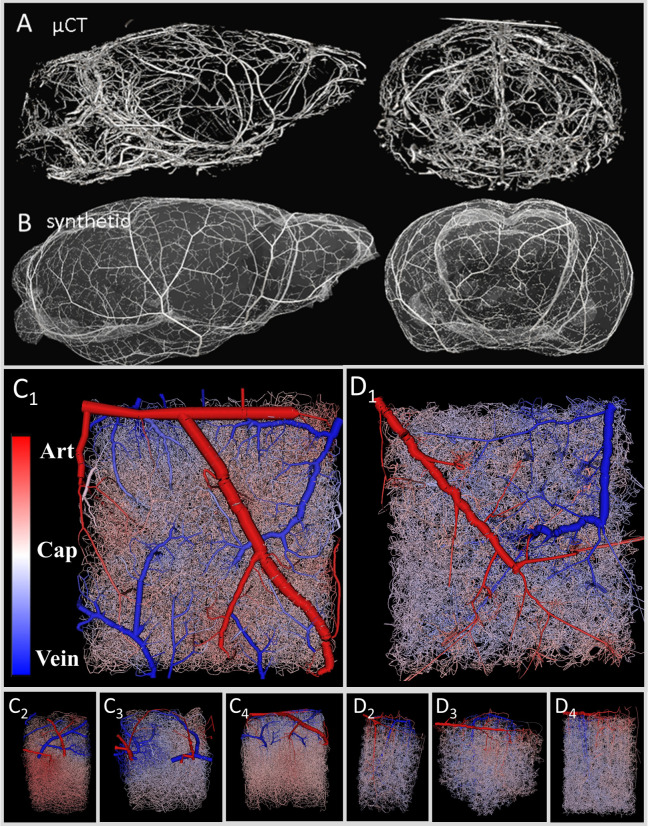
Multi-modal imaging data used to construct realistic models of cerebral circulation for entire mouse brain. (A,B) *Main blood vessels and pial arterial network*. (A) High resolution µCT image of the vascular tree in mouse. (B) Synthetic pial arterial tree generated by modified constrained constructive optimization and morphological data from the µCT images. Note that the synthetic circulatory network does not perfectly reproduce the layout of the pial vessels in the µCT, but merely possesses similar morphometrics. (C,D) *Cortical microcirculation* (C_1_-C_4_) Experimental microcirculatory networks acquired with two-photon laser imaging [35]. (D_1_-D_4_) Synthetic microcirculatory data sets. The synthesized data sets are statistically equivalent to the reconstructed networks from 2PLSM. Arterial (red) and venous side (blue) are color coded for better visibility.

An overview of the data structures used in this study is presented in Fig 9.

### Pial surface data acquisition

Nine female C57BL/6 mice were imaged for pial vascular network structures following intravascular injection of a lead pigment contrast agent as described elsewhere [56,63–65]. The mice were perfusion fixed prior to micro computed tomography (µCT) imaging with 7–20 µm isotropic resolution of the cerebral angioarchitecture. The resulting 3D images were filtered and the vascular lumen reconstructed as previously described [66–68]. Fig 9A shows raw µCT samples of the mouse vasculature from a 20 µm resolution image. The pial network statistics such as penetrating arteriole density and vessel diameter were compiled with results summarized in Table 2.

### Microcirculatory data acquisition

Four volumes (N = 4) that encompassed the murine vibrissa primary sensory cortex [35] were imaged using two-photon laser scanning microscopy (2PLSM) and are shown in Fig 9C. 2PLSM was employed to extract the spatial arrangement, length and orientation of blood vessels in the vibrissa primary sensory cortex [31,35,69]. Blood vessels in four data sets (~1x1x1 mm^3^) were labeled as pial arteries, penetrating arterioles, capillaries, ascending venules, or pial veins. Categorization was based on size and branching level according to Strahler order rather than physiological markers. No effort was made to differentiate pre-capillary arterioles from post-arteriole capillaries because it requires differential labeling of smooth muscle and pericytes. Capillaries were differentiated from ascending venules by a diameter cutoff of 6 µm and penetrating venules were differentiated from pial veins for vessels within a depth of 100 µm below the pia and a diameter less than 12 µm. Diameter information was also derived from images. The network information was stored using sparse connectivity matrices. Length, diameter, and tortuosity spectra are depicted in Fig 1. More details on image acquisition [31,35,69], image reconstruction [70], as well as the formulation of the network equations [29] can be found elsewhere.

### Synthesis of large circulatory networks

Artificial microvascular networks (N = 60) for large sections of the cortex (~1x1x1 mm^3^) were synthesized using a previously described vascular growth algorithm [30]. Four examples are displayed in Fig 9D. The algorithm preserved dimensions of the experimentally acquired cortical samples, pattern and dimension of pial arteries, number, orientation and connectivity of penetrating arterioles, and morphometrics of the capillary bed, draining venules and pial veins, as listed in Table 1. Statistics and morphometric comparisons of experimental and synthetic data sets are displayed in Fig 1.

The arterial network of the entire MCA territory spanning three orders of magnitude in length from large arteries (~1 mm range) down to the entire capillary bed (~1 µm) was synthesized based on morphometric statistics of source data from multimodal images (µCT and 2PLSM).

### Blood flow

Microcirculatory blood flow was modeled as a biphasic suspension comprised of red blood cells and plasma. Bulk blood flow was described by Poiseuille law relating volumetric flow to pressure drop as a function of resistance which in turn depends on diameter, d, and hematocrit-dependent viscosity [71]. In addition, a kinetic plasma skimming model (KPSM) presented previously [29] accounted for the uneven RBC distribution, known as plasma skimming.

This model has only one adjustable parameter, the skimming coefficient, m. It was set to value of m = 8 in all microcirculatory models, although this parameter could be refined as shown recently [72–74]. The nonlinear systems of conservation balances in system (1) were solved iteratively to calculate blood pressures, p, flow, Q, and hematocrit, h. Here, R is the resistance matrix, C_1_ and C_2_ are fundamental connectivity matrices [75] and C_3_ is the advection flux matrix. Boundary conditions are summarized in Table 3. More details on the mathematical background are given in S2 Supplement; implementation details are discussed elsewhere [29].


$$G(Q,p,h)=0\left\{ \begin{array}{cc} R(h,d)Q-C_{1}p & =0\\ C_{2}Q & =0\\ C_{3}(Q,d)h & =0 \end{array}\right.$$ (1)

**Table 3:** Summary of boundary conditions.

Arterial Inlet	*p* = 120 mmHg (15,999 Pa)
Venous Outlet	*p* = 5 mmHg (667 Pa)
Inlet Hematocrit	*h* = 0.35
Outlet Hematocrit	Fully developed, ∇*h* = 0
lower boundaries (GM/WM interface)	confined domain (zero flux, ∇Q = 0)
sides boundaries (vertical tissue boundary)	cyclic boundary conditions or confined domain (zero flux, ∇Q = 0)

## Supporting information

S1 Supplement Hematocrit dependence on diameter. (DOCX)

S2 Supplement Implementation of biphasic blood flow. (DOCX)

S3 Supplement Hematocrit split rules. (DOCX)
