# Supplementary material for: Simulations of blood as a suspension predicts a depth dependent hematocrit in the circulation throughout the cerebral cortex
Source: PLoS Comput Biol. 2018 Nov 19;14(11):e1006549. doi: 10.1371/journal.pcbi.1006549 (PMC6277127; doi:10.1371/journal.pcbi.1006549)
Supplement: S1 Supplement — (DOCX) [file pcbi.1006549.s001.docx]

**S1 Supplement: Hematocrit dependence on diameter**

This section plots hematocrit distribution as a function of diameter. Fig A in S1 Supplement reflects wide variability in discharge hematocrit levels. Path analysis discussed in the main paper in Fig 2 and Fig 3 revealed a depth-dependent trend in hematocrit distribution independent of diameter.

| 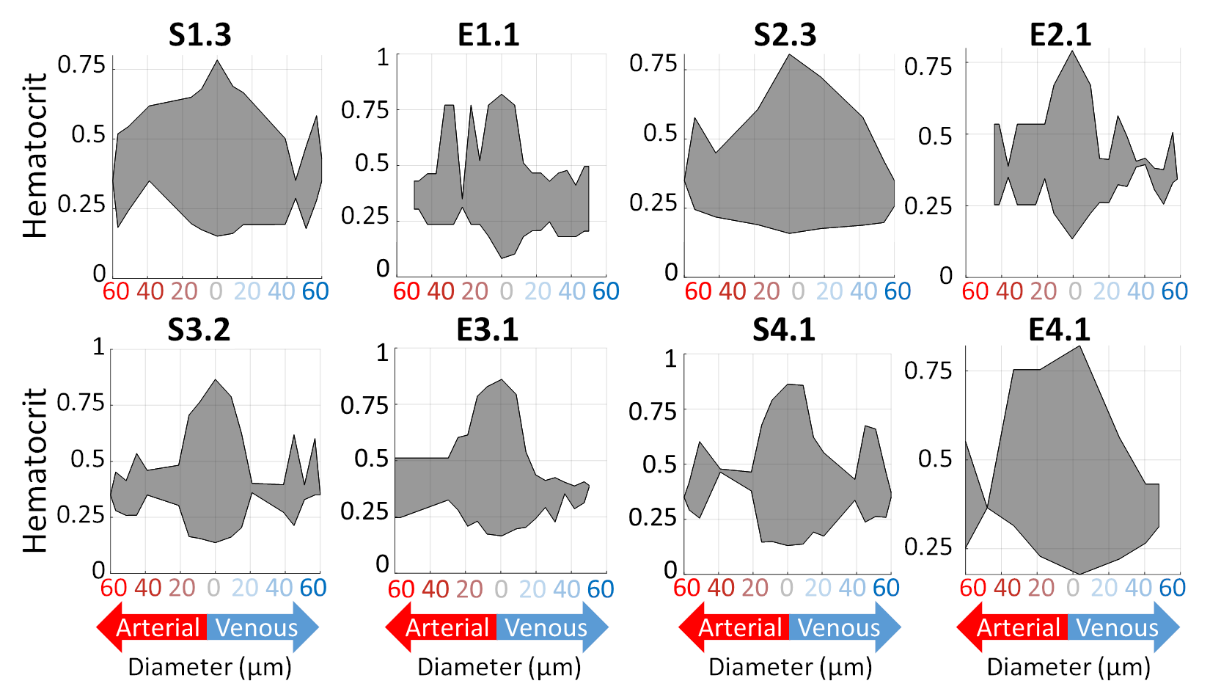 |
| --- |
| **Fig A: Diameter dependence of hematocrit in microcirculatory networks.** The analysis confirms the high degree of hematocrit variability along the microcirculatory bed. Diameter hierarchy did not correlate with hematocrit level (N = 2,300-22,052 paths per dataset). |
