# Supplementary material for: Simulations of blood as a suspension predicts a depth dependent hematocrit in the circulation throughout the cerebral cortex
Source: PLoS Comput Biol. 2018 Nov 19;14(11):e1006549. doi: 10.1371/journal.pcbi.1006549 (PMC6277127; doi:10.1371/journal.pcbi.1006549)
Supplement: S2 Supplement — (DOCX) [file pcbi.1006549.s002.docx]

**S2 Supplement: Implementation of the biphasic blood flow**

Biphasic blood flow was solved computationally by enforcing three conservation laws: conservation of mass, linear momentum, and RBC fluxes using a plasma skimming model. For the RBC splitting rule, we used the KPSM model [1] with a constant plasma skimming coefficient, *m*, although the accuracy could be further improved by adjusting the *m*-value as a function of hematocrit and diameter as shown by Yang et al. [2–4].

The system of conservation laws in Eq. (1) in S2 Supplement is highly nonlinear and coupled. We showed previously a beneficial decomposition technique that enables the consecutive but separate convergence of linear algebraic subsystems [1]. The advantages of our implementation include the ability for each subsystem to be solved separately with highly efficient sparse iterative linear algebraic solvers. Additionally, no derivative information is necessary. In effect, we perform the fixed point iteration algorithm depicted in Fig A in S2 Supplement. Given an initial hematocrit field (initially set to systemic hematocrit *h* = 0.35), solve for the pressure in the first equation set using the resistance matrix, 𝑅, which incorporates the nonideal hematocrit dependent viscosity law. With the converged pressure field, 𝑝, compute the flow field, Q, using the connectivity matrix 𝐶_2_. Finally, update the hematocrit field, ℎ, using the kinematic plasma skimming (KPSM) law to determine how the hematocrit in parent segments splits into two or more daughter branches. In order to limit the magnitude of hematocrit updates, we use an underrelaxation parameter α=0.5 for stabilization. More details on implementation and stabilization can be found elsewhere [1].

| $G\left( Q,p,h \right)= 0\left\{ \begin{matrix} R\left( h,d \right)Q-C_{1}p & =0 \\ C_{2}Q & =0 \\ C_{3}\left( Q,d \right)h & =0 \end{matrix} \right.$ | (1) |
| --- | --- |

| 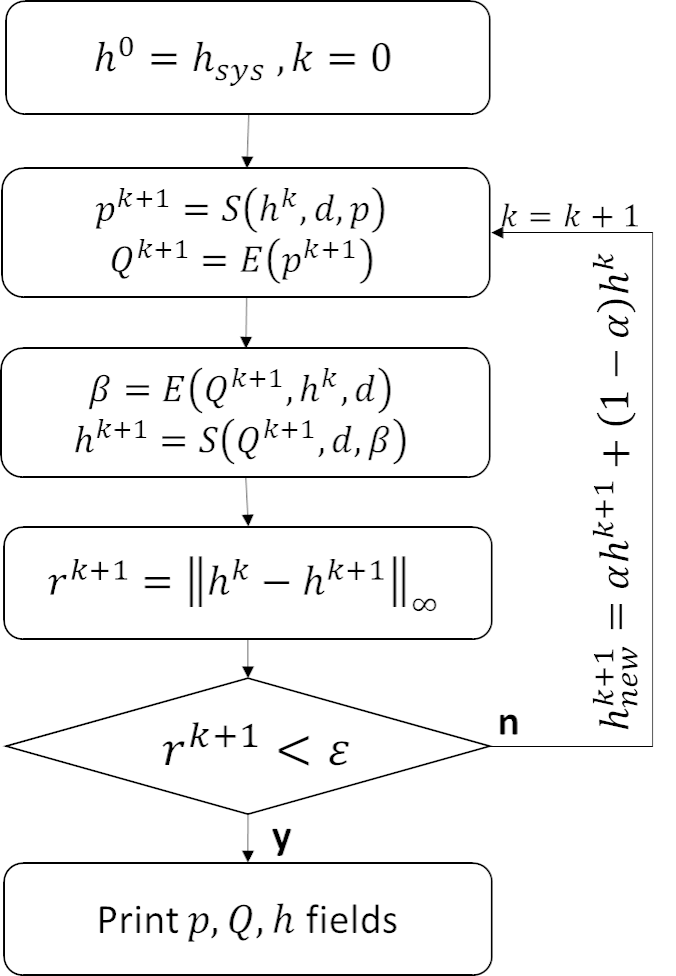 |
| --- |
| **Fig A: Flow diagram for main steps in the fixed point iteration for biphasic blood flow computations.** Here, 𝐸 means an evaluation step; S signifies simultaneous solution of linear algebraic equation sets. |

We also implemented cyclical boundary conditions at side faces perpendicular to the pial surface. This was achieved by connecting boundary segments located near one vertical face of the domain to a corresponding node at an opposite face. Matching boundary blood vessel facets in all cortical layers, the domain boundaries were effectively extended to infinity, without losing blood flow or RBCs. We also implemented no flux boundary conditions and compared the results to cyclic boundary conditions. Because of the large domain size and similar conditions governing opposite faces, simulation results between those two choices were very close (total flow differences less than 0.0001%).

**Bibliography**

1. Gould IG, Linninger AA. Hematocrit distribution and tissue oxygenation in large microcirculatory networks. Microcirculation. 2015 Jan 1; 22(1):1–18.

2. Yang J, Yoo SS, Lee T-R. Effect of fractional blood flow on plasma skimming in the microvasculature. Phys Rev E. 2017 Apr 25; 95(4):040401.

3. Yang J, Pak YE, Lee T-R. Predicting bifurcation angle effect on blood flow in the microvasculature. Microvasc Res. 2016 Nov 1;108:22–8.

4. Lee T-R, Yoo SS, Yang J. Generalized plasma skimming model for cells and drug carriers in the microvasculature. Biomech Model Mechanobiol. 2017 Apr 1; 16(2):497–507.
