# Supplementary material for: Simulations of blood as a suspension predicts a depth dependent hematocrit in the circulation throughout the cerebral cortex
Source: PLoS Comput Biol. 2018 Nov 19;14(11):e1006549. doi: 10.1371/journal.pcbi.1006549 (PMC6277127; doi:10.1371/journal.pcbi.1006549)
Supplement: S3 Supplement — (DOCX) [file pcbi.1006549.s003.docx]

**S3 Supplement - Hematocrit split rules**

We first attempted to make use of a hematocrit splitting rule for biphasic blood flow computations derived from experiments in the rat mesentery. At first, we tried three different versions of the Secomb-Pries split rule developed for small networks of the rat mesentery which do not exhibit multifurcations, and for which numerous different parameter choices can be found in the literature [1–5]. However, none of the different versions can be applied to in vivo data sets with multifurcations (654, 725, 1868, and 1440 in the four experimentally-derived microcirculatory networks). We also tried to simulate biphasic blood flow of two midsize network of a cortical microcirculation with about 2000 and 10,000 splined segments, see Fig A in S3 Supplement and Table A in S3 Supplement. However, all simulations with the Secomb-Pries splitting rule contained segments with physiologically problematic results. Table A in S3 Supplement summarizes convergence results and problematic predictions by their splitting rule. For the large-scale MCA blood flow simulations, the Secomb-Pries model rapidly diverged in all simulation runs.

| 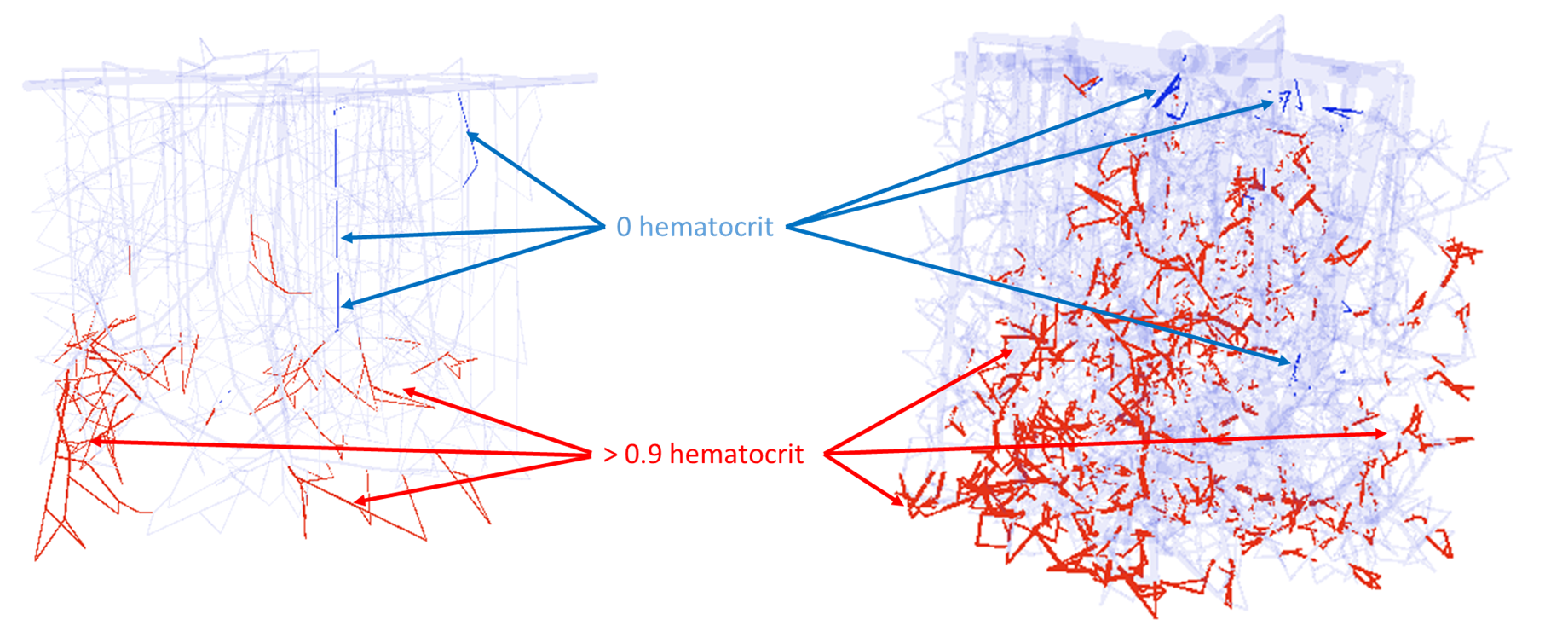 |
| --- |
| **Fig A. Hematocrit fields portrayed on two simplified microcirculatory models.** Obtained with the Pries 2005 splitting rule [1] showing many segments with non-physiological results. Many segments had zero RBC flux (Hct ~0), other segments reached hematocrit levels that fell outside the definition space of the hematocrit-dependent viscosity (Hct > 0.9). Red indicates excessive hematocrit and dark blue indicates vessels with zero RBC flux. Taken together, these data and more results listed in Table A explain why the Pries splitting rule, with any combination of parameters, was not applicable to simulate large-scale network effects in the mouse cortex. Instead, the KPSM model with implementation in Appendix A was used which gave consistently, physiologically meaningful results in all cases as listed in Table A. |

*Zero RBC flow*. For example, the 2005 version [1] had numerous segments without RBCs. The 2011 version [1] and the 2015 version [2] did not converge for larger networks (Table A in S3 Supplement).

*Excessive RBC flow*. Moreover, all predictions based on the Pries family of models [1–3] led to segments with excessive discharge hematocrit greater than 0.9 (*h* > 0.9). This value is outside the validity range of the viscosity law, which is only valid [6–9] for hematocrit up to up to 0.7 (*h*
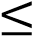
 0.7). The 2005 version [1] of coefficients had up to 11.8% vessels above the excessive hematocrit threshold. Numerical complications were also noted in a study [1] which deployed the Pries split model, stating the following comments on the splitting rule:

“… a threshold of 0.8 for the hematocrit in the daughter branches was prescribed …”

“… hematocrit was set to zero in daughter branches with flow below a given threshold …”

In a recent report [5], the authors retorted our earlier findings [10] about the inconsistencies of their split rules. They updated their formulae with a new set of three parameters in their highly non-linear functions to reduce the number of occurrences of RBC free vessels. The effort to eliminate RBC free vessels stands in contrast to another recent review [11] which suggests that zero RBC predictions are a necessary feature as expressed in the following quote:

“This approach (KPSM model by Gould et al, 2015) excludes the possibility of zero hematocrit in low-flow branches, whereas such behavior can be observed in vivo…”

In our view, zero RBC flow prediction for a stationary simulation is not physiologically meaningful. The prediction of RBC free blood flow points to a structural problem of the empirical split rule by Pries that cannot be remedied by parameters adjustments. Inspection of the split formula shows a discontinuous “if-statement” which sets one branch of the Secomb-Pries split formula artificially to zero to avoid transgressing the definition space of the *logit* function.

We have no further comment on the applicability of the Secomb-Pries split rule for single bifurcations or small mesentery networks. However, simulations for large cerebral microcirculatory networks as shown in this and in prior studies [10,12] point to two principal reasons why the KPSM model was chosen:

- First, the KPSM model can address multifurcations which occur in realistic data sets (654, 725, 1868, and 1440 in four vibrissa primary sensory cortex networks), while the Secomb-Pries models in all their variants cannot.
- Second, the implementation of the KPSM model with a single parameter, m, predicts hematocrit distribution within physiological ranges for large cerebrocirculatory networks.

Table A: Physiological assessment of plasma skimming models

|  | Segments with h=0 | | | Segments with discharge h > 0.9 | | |
| --- | --- | --- | --- | --- | --- | --- |
| **Simplified Microcirculation 1** |  |  |  |  |  |  |
| **KPSM** | ✓ | ✓ | ✓ | ✓ | ✓ | ✓ |
| Pries [1] 2005 | ● | ✓ | 2 | ● | 231 | 217 |
| Lorthois et. al [2] 2011 | ● | ● | ● | ● | ● | ● |
| Chebbi et. al [3] 2015 | ● | ● | ● | ● | ● | ● |
| **Simplified Microcirculation 2** |  |  |  |  |  |  |
| **KPSM** | ✓ | ✓ | ✓ | ✓ | ✓ | ✓ |
| Pries [1] 2005 | 316 | ● | 54 | 1397 | ● | 2159 |
| Lorthois et. al [2] 2011 | ● | ● | ● | ● | ● | ● |
| Chebbi et. al [3] 2015 | ● | ● | ● | ● | ● | ● |
| **Massive MCA Simulation** |  |  |  |  |  |  |
| **KPSM** | ✓ | ✓ | ✓ | ✓ | ✓ | ✓ |
| Pries [1] 2005 | ● | ● | ● | ● | ● | ● |
| Lorthois et. al [2] 2011 | ● | ● | ● | ● | ● | ● |
| Chebbi et. al [3] 2015 | ● | ● | ● | ● | ● | ● |

**V1** = Pries In Vitro, V2 = Pries In Vitro Modified, V3 = Pries In Vivo, ●=not converged, ✓=converged without faces (h~0, h>0.9, h discharge hematocrit)

**Bibliography**

1. Pries AR, Secomb TW. Microvascular blood viscosity in vivo and the endothelial surface layer. Am J Phys - Heart C. 2005 Dec 1; 289(6):H2657–64.

2. Lorthois S, Cassot F, Lauwers F. Simulation study of brain blood flow regulation by intra-cortical arterioles in an anatomically accurate large human vascular network: Part I: methodology and baseline flow. Neuroimage. 2011 Jan 15; 54(2):1031–42.

3. Chebbi R. Dynamics of blood flow: modeling of the Fåhræus–Lindqvist effect. J Biol Phys. 2015 Jun 1; 41(3):313–26.

4. Pries AR, Ley K, Claassen M, Gaehtgens P. Red cell distribution at microvascular bifurcations. Microvasc Res. 1989 Jul 1; 38(1):81–101.

5. Rasmussen PM, Secomb TW, Pries AR. Modeling the hematocrit distribution in microcirculatory networks: A quantitative evaluation of a phase separation model. Microcirculation. 2018 Mar 10; 20(2):e12445.

6. Klitzman B, Duling B. Microvascular hematocrit and red cell in resting and contracting striated muscle. Vol. 237. 1979; H481-H490.

7. Lipowsky HH, Usami S, Chien S. In vivo measurements of “apparent viscosity” and microvessel hematocrit in the mesentery of the cat. Microvascular Research. 1980 May 1; 19(3):297–319.

8. Pries AR, Secomb TW, Gessner T, Sperandio MB, Gross JF, Gaehtgens P. Resistance to blood flow in microvessels in vivo. Circulation Research. 1994 Nov 1; 75(5):904–15.

9. Tang Z, Lee JH. Effects of different hematocrit levels on glucose measurements with handheld meters for point-of-care testing. Arch Pathol Lab Med. 2000; 124:6.

10. Gould IG, Linninger AA. Hematocrit distribution and tissue oxygenation in large microcirculatory networks. Microcirculation. 2015 Jan 1;22(1):1–18.

11. Gagnon L, Smith AF, Boas DA, Devor A, Secomb TW, Sakadžić S. Modeling of cerebral oxygen transport based on in vivo microscopic imaging of microvascular network structure, blood flow, and oxygenation. Front Comput Neurosci. 2016;10.

12. Gould IG, Tsai P, Kleinfeld D, Linninger A. The capillary bed offers the largest hemodynamic resistance to the cortical blood supply. J Cereb Blood Flow Metab. 2017 Jan 1; 37(1):52–68.
